# Efficacy of geraniin on dengue virus type-2 infected BALB/c mice

**DOI:** 10.1186/s12985-019-1127-7

**Published:** 2019-02-27

**Authors:** Siti Aisyah Abdul Ahmad, Uma D. Palanisamy, Joon Joon Khoo, Amreeta Dhanoa, Sharifah Syed Hassan

**Affiliations:** 1 Jeffrey Cheah School of Medicine and Health Sciences, Monash University Malaysia, Jalan Lagoon Selatan, 47500 Bandar Sunway, Selangor Malaysia; 2 Clinical School Johor Bahru, Jeffrey Cheah School of Medicine and Health Sciences, Monash University Malaysia, 8, Jalan Masjid Abu Bakar, 80100 Johor Bahru, Johor Malaysia; 3 Infectious Diseases and Health Cluster, Tropical Medicine and Biology Platform, Monash University Malaysia, Jalan Lagoon Selatan, 47500 Bandar Sunway, Selangor Malaysia

**Keywords:** Antiviral, Dengue virus, Geraniin, In vivo

## Abstract

**Background:**

Dengue continues to be a major international public health concern. Despite that, there is no clinically approved antiviral for treatment of dengue virus (DENV) infections. In this study, geraniin extracted from the rind of *Nephelium lappaceum* was shown to inhibit the replication of DENV-2 in both in vitro and in vivo experiments.

**Methods:**

The effect of geraniin on DENV-2 RNA synthesis in infected Vero cells was tested using quantitative RT-PCR. The in vivo efficacy of geraniin in inhibiting DENV-2 infection was then tested using BALB/c mice with geraniin administered at three different times. The differences in spleen to body weight ratio, DENV-2 RNA load and liver damage between the three treatment groups as compared to DENV-2 infected mice without geraniin administration were determined on day eight post-infection.

**Results:**

Quantitative RT-PCR confirmed the decrease in viral RNA synthesis of infected Vero cells when treated with geraniin. Geraniin seemed to provide a protective effect on infected BALB/c mice liver when given at 24 h pre- and 24 h post-infection as liver damage was observed to be very mild even though a significant reduction of DENV-2 RNA load in serum was not observed in these two treatment groups. However, when administered at 72 h post-infection, severe liver damage in the form of necrosis and haemorrhage had prevailed despite a substantial reduction of DENV-2 RNA load in serum.

**Conclusions:**

Geraniin was found to be effective in reducing DENV-2 RNA load when administered at 72 h post-infection while earlier administration could prevent severe liver damage caused by DENV-2 infection. These results provide evidence that geraniin is a potential candidate for the development of anti-dengue drug.

## Background

Dengue is a debilitating disease spread through the bite of an *Aedes* mosquito that carries DENV. The four serotypes of DENV (DENV-1, DENV-2, DENV-3 and DENV-4) belong to the genus *Flavivirus* and the family *Flaviviridae* [1]. DENV genome is made up of a single stranded positive-sense RNA which encodes three structural (capsid [C], membrane [M], and envelope [E]) and seven non-structural (NS1, NS2A, NS2B, NS3, NS4A, NS4B, and NS5) proteins [2]. Infection with DENV causes a wide spectrum of clinical manifestations ranging from undifferentiated fever, classical dengue fever to severe sometimes fatal manifestation characterized by plasma leakage with or without haemorrhage [3]. Almost 75% of the global population exposed to dengue live in Asia-Pacific region, with 1.3 billion of these at-risk individuals living in Southeast Asia region [4]. Despite the advancement of today’s drugs discovery and development, no effective anti-dengue drug has been approved for treatment of DENV infections with meticulous fluid management remaining the mainstay of treatment.

The absence of an appropriate animal model that can depict the true nature of the complex dengue pathogenesis has contributed to our lack of understanding of its pathogenesis, which is crucial in the process of developing any vaccines or antivirals [5]. This absence has hindered research on dengue, especially on how the viral and host factors contributed to the severe forms of this disease [6]. The only known natural hosts for DENV are humans and mosquitoes. When wild-type mice and other non-human primates were used as the animal model for DENV infection, clinical isolates of DENVs showed a low level or lack of viral replication and clinical disease [7]. Paes and co-workers [8] had tested the feasibility of 8 weeks old male BALB/c mice as an experimental model in the study of dengue disease. They reported that DENV-2 infected BALB/c mice developed an apparently mild infection, but histopathological and biochemical findings revealed liver injury. The presence of DENV-2 was confirmed when viral antigens were detected in focal areas of the damaged liver, thus making it an ideal model to investigate the pathogenesis of dengue disease and henceforth the anti-dengue potentials of natural compounds.

**Fig. 1:**
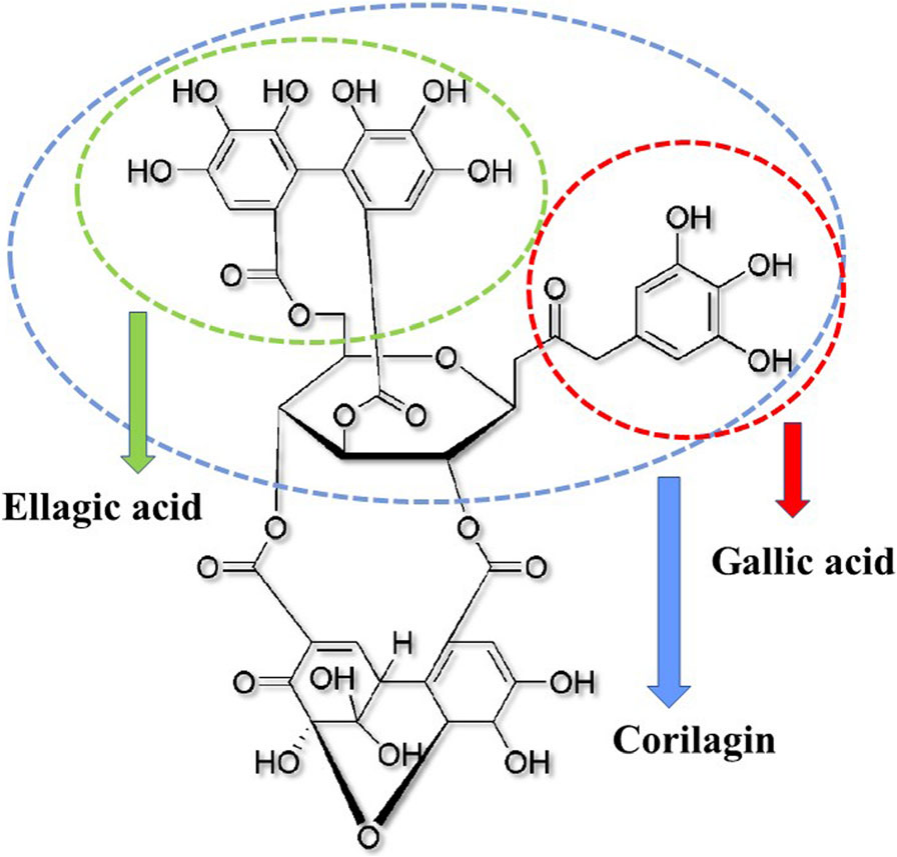
Chemical structure of geraniin. Geraniin is a hydrolysable tannin with various physical properties. Upon hydrolysis, geraniin will be broken down into smaller metabolites such as gallic acid, ellagic acid and corilagin. The chemical structures of the metabolites are as circled

Previous studies have proven that several natural compounds are effective in inhibiting DENV infection and have the potential to be developed as anti-dengue drugs [9–13]. Geraniin is an ellagitannin, a type of polyphenolic compound with a chemical formula of C_41_H_28_O_27_ and a molecular mass of 952.64 g/mol, that is widely distributed in nature and has various medicinal properties [14]. Geraniin was reported to exhibit various antiviral, anti-inflammatory, apoptotic, cytotoxicity against cancer cells, cytoprotective, antimicrobial and antioxidant properties [15]. In physiological conditions, geraniin has been known to hydrolyse to form corilagin, ellagic acid and gallic acid [16]. Structure of geraniin and its metabolites are shown in Fig. 1. Studies have shown that geraniin, as well as its metabolites have antiviral activities against several types of viruses. Geraniin isolated from other plants have been shown to exhibit antiviral properties against herpes simplex virus [17], human immunodeficiency virus [18], hepatitis B virus [19], and human enterovirus 71 [20]. Interestingly, a medicinal cocktail containing four *Phyllanthus sp*, was shown to possess inhibitory activity against DENV-2, with more than 90% virus reduction [21]. While geraniin was identified as one of the active compounds in this cocktail, there was no evidence indicating that geraniin was indeed the compound contributing to the reduction in viral load.

The rationale for antiviral treatment in DENV infections arises from clinical studies showing an association between higher quantity of circulating virus in the first few days of illness and severe disease outcome, and longer duration of viremia in severe cases [22, 23]. In addition to high viremia titre, increased dengue severity has also been linked to secondary DENV infection and DENV-2 serotype [24]. These findings support the clinical benefit of antiviral drugs, that is to reduce the viral load in the acute phase of the disease. A suitable antiviral against DENV may be used therapeutically to stop progression towards severe dengue [5].

Many natural products that showed good inhibitory potential against certain viruses in vitro have failed to progress to the clinical trials phase because of its poor activity in vivo. It is stated that a natural product or its purified component that has strong antiviral activity in vitro but no therapeutic efficacy against virus infection in an animal infection model is merely an inhibitor and not a medicine [25]. Thus, in this study, in vivo inhibition of DENV-2 by geraniin was investigated. This study is the first to investigate both in vitro and in vivo anti-dengue potential of geraniin, in Vero cells and male BALB/c mice respectively. Geraniin used in this study was purified from the rind of *Nephelium lappaceum* [26]. *Nephelium lappaceum*, or locally known as rambutan, is a type of fruit which can be found in abundance in tropical countries such as Malaysia, Indonesia, Philippines, Thailand, and several other regions of the Southeast Asia. This study also investigates the prophylactic ability of geraniin (treatment given 24 h before infection), as well as its therapeutic effect (treatment given at 24 and 72 h post-infection) on DENV-2 infected BALB/c mice.

## Methods

### Cell culture and virus

Propagation of DENV-2 was conducted in the African Green Monkey kidney cells (Vero) (ATCC CCL-81). The cells were maintained at 37 °C with 5% CO_2_ and propagated in Minimum Essential Medium (MEM) supplemented with 10% foetal bovine serum (FBS), HEPES buffer and penicillin/streptomycin (Gibco®; Life Technologies, Carlsbad, CA, USA). DENV-2 (accession no: AJ556810.1) was kindly provided by Prof. Sazaly AB (University of Malaya, Malaysia). Infected cells were frozen and thawed three times before they were harvested and centrifuged at 4500 RPM for 30 min at 4 °C. Supernatant containing viruses were filtered using 0.2 μm syringe filter (Merck Millipore; Burlington, MA, USA) into a new sterile bottle and were mixed with sterilized 21% PEG/6.9% NaCl and stirred overnight at 4 °C. The homogenized mixture was then centrifuged at 12,000 RPM for 40 min at 4 °C. The pellet was resuspended in TNE buffer followed by overnight dialysis at 4 °C. Sucrose gradient was then conducted using ultracentrifuge (Beckman coulter; Brea, CA, USA) at 34,400 RPM for 8 h at 4 °C. A single band in between the sucrose layers containing pure DENV-2 was collected. Desalting of sucrose from the virus was conducted using Amicon Ultra Centrifugal filter (Merck Millipore). The viral titre of the purified virus was determined using the TCID_50_ method [27]. Pure DENV-2 in phosphate buffered saline (PBS) was then stored in − 80 °C until further use.

### Compound

Geraniin was isolated and purified from the rind of *Nephelium lappaceum* as described by Perera et al. [26]. Geraniin of purity above 97% was used in this study. Geraniin was initially dissolved in 0.2% dimethyl sulfoxide (DMSO; Sigma-Aldrich; St. Louis, MO, USA) and diluted to the required concentration immediately prior to the experiments either in MEM containing 2% FBS or in PBS for in vitro and in vivo experiments respectively. For in vitro experiment, control cells were treated with MEM supplemented with a final concentration of 0.2% DMSO while for in vivo experiment, control mice were administered with geraniin initially dissolved in 0.2% DMSO.

### Antiviral assay of geraniin by quantitative RT-PCR

Vero cells were seeded into 24-well-plate at a density of 2 × 10^5^ cells/ml and were incubated overnight. DENV-2 with a titre of 2 × 10^4^ TCID_50_/ml was mixed with or without different concentrations of geraniin and were simultaneously adsorbed onto cells for 1 h at 37 °C. The virus-geraniin mixture were removed and replaced with fresh medium and the cells were incubated for another 48 h. The cell monolayer was collected, and total intracellular virus RNA was extracted using the TRI Reagent® Solution (Molecular Research Center, Inc.; Cincinnati, OH) as instructed by the manufacturer. A two-step quantitative RT-PCR (qRT-PCR) was then performed. First, cDNA was synthesized using the Maxima First Strand cDNA Synthesis Kit (Fermentas, Thermo Fisher Scientific Inc.; Waltham, MA, USA); second, amplifications were carried out using the KAPA SYBR® FAST qPCR Master Mix (Kapa Biosystems; Woburn, MA, USA) with the primer pairs designed from conserved region of the DENV-2 E gene: forward (DENV2EFa) 5′-GGCCTCGACTTCAATGAGATGG-3′ (1482–1503), reverse (DENV2ERa) 5′-CCTGTTTCTTCGCATGGGGAT-3′ (1639–1659 complement) on the ABI Step One and Step One Plus Real Time PCR Systems (Applied Biosystems; Foster City, CA, USA). Cycles consisted of an initial incubation step at 94 °C for 2 min, 40 cycles at 94 °C for 30 s, 60 °C for 30 s, and 72 °C for 30 s, and a melting curve analysis cycle. Quantification of DENV-2 RNA load was obtained using the standard curve method. The standard curve was generated using serial 10-fold dilutions of the stock DENV-2 RNA with known amount of virus. The percentage of DENV-2 RNA level reduction in geraniin treated cells relative to the untreated control was calculated to construct a dose-response curve and the IC_50_ value was obtained using the GraphPad Prism 5 software.

### Treatments of infected BALB/c mice with geraniin

The ethical approval for animal experiments was approved by the Monash University Animal Ethics Committee (AEC) MARP/2015/069. Groups of six male BALB/c mice, aged between six to eight weeks were divided into six groups; (1) uninfected and untreated [Control A], (2) uninfected and geraniin treated [Control B], (3) Untreated DENV-2 infected [Infected A], (4) DENV-2 infected and treated with geraniin prophylactically 24 h prior to infection [Infected B], (5) DENV-2 infected and treated with geraniin 24 h post-infection (p.i.) [Infected C] and (6) DENV-2 infected and treated with geraniin 72 h p.i. [Infected D]. DENV-2 infection was achieved by inoculating with 2 × 10^4^ TCID_50_/mouse DENV-2, while geraniin treatment was with 131.30 μM prepared in 100 μl PBS. Both DENV-2 and geraniin were intravenously administered into each mouse via the tail vein. The group “Control B” served to rule out toxicity of geraniin at the dosage used. The mice were monitored daily for any development of clinical signs until the completion of the study. At the end of the 8th day, the weights of mice were recorded before they were anaesthetized with a mixture of ketamine-xylazine and sacrificed by cardiac puncture. Livers were collected for histopathological analysis while spleens were collected to observe for any changes in size. The weight of each spleen and its corresponding body weight was recorded for the spleen-to-body weight ratio.

### Determination of DENV-2 RNA load in serum

At 8 days p.i., blood samples were collected by terminal cardiac puncture (approximately 700 μl). Serum samples were obtained by centrifugation at 2000 RPM for 15 min at 4 °C after blood coagulation. RNA extraction was carried out using the TRI Reagent according to manufacturer’s instructions. Reverse transcription was then carried out with Maxima First Strand cDNA Synthesis Kit (Thermo Fisher Scientific) to obtain first strand cDNA. RT-PCR was performed under the following conditions: 1 μl 10X dsDNase buffer, 1 μl dsDNase, 100 ng/μl template RNA and water were mixed and incubated for 2 min at 37 °C. The reaction was chilled on ice followed by the addition of 4 μl 5X reaction mix, 2 μl Maxima enzyme mix, and 4 μl water and incubated for 10 min at 25 °C followed by 15 min at 50 °C. Lastly, the reaction was terminated by heating at 85 °C for 5 min. The cDNA product was then used directly in qPCR. The SYBR Green based qPCR was performed using KAPA SYBR FAST qPCR Master Mix (Kapa Biosystems) and the primer pair DENV2EFa and DENV2Ra. Quantification of DENV-2 RNA was obtained using the standard curve method on the ABI Step One and Step One Plus Real Time PCR Systems (Applied Biosystems). The standard curve was generated using serial 10-fold dilutions of a plasmid containing DENV-2 gene.

### Haematoxylin and eosin staining

Upon the sacrifice of the animals at 8 days p.i., the skin of each mouse was cleaned with 70% isopropanol and their livers were dissected using sterile surgical instruments. These livers were fixed in 10% formaldehyde and sent to University Putra Malaysia (UPM) for haematoxylin and eosin staining. Slides were analysed for histopathological changes and semi-quantitative assessment of different histological parameters for each treatment group was performed. Table 1 shows the subjective scale used for the semi-quantitative analysis, with detailed descriptions of the evaluated histopathological alterations. Scoring for the semi-quantitative analysis of liver sections from six replicates mice of each treatment group was conducted based on the subjective scale.

**Table 1:** Subjective scale for semi-quantitative analysis of the degree of liver injury based on histopathological alterations

Histopathological alterations	0	1	2	3
Hydropic degeneration (hepatocyte swelling)	None	Mild (small area, ground glass appearance of hepatocytes)	Moderate (larger area, ground glass appearance of hepatocytes)	Diffuse (feathery appearance of hepatocytes)
Necrosis & haemorrhage	None	Mild (no haemorrhage)	Moderate (small area, focal)	Severe (large area, multifocal)
Venous outflow congestion (dilatation of sinusoids)	None	Mild (slight)	Moderate (focal)	Severe (diffuse)
Steatosis	None	Mild (small area, microsteatosis)	Moderate (larger zones, micro- and macrosteatosis)	Severe (diffuse micro- and macrosteatosis)
Pyknotic nucleus	None	Mild	Moderate	Severe

Liver sections from six replicates mice (n = 6) were evaluated using a scale ranging from 0 to 3 (0 = none, 1 = mild, 2 = moderate and 3 = severe) according to the degree of hydropic change (hepatocyte swelling); necrosis and haemorrhage; venous outflow congestion (dilatation of sinusoids); steatosis; and pyknotic nucleus

### Statistical analysis

The software GraphPad Prism (Version 5.0; La Jolla, CA, USA) was used for all statistical analysis and graphical illustrations. Statistical analysis consisted of one-way ANOVA followed by Dunnett’s multiple comparison test, with a significance of *P* < 0.05.

## Results

### Inhibitory effect of geraniin on intracellular DENV-2 RNA synthesis

**Fig. 2:**
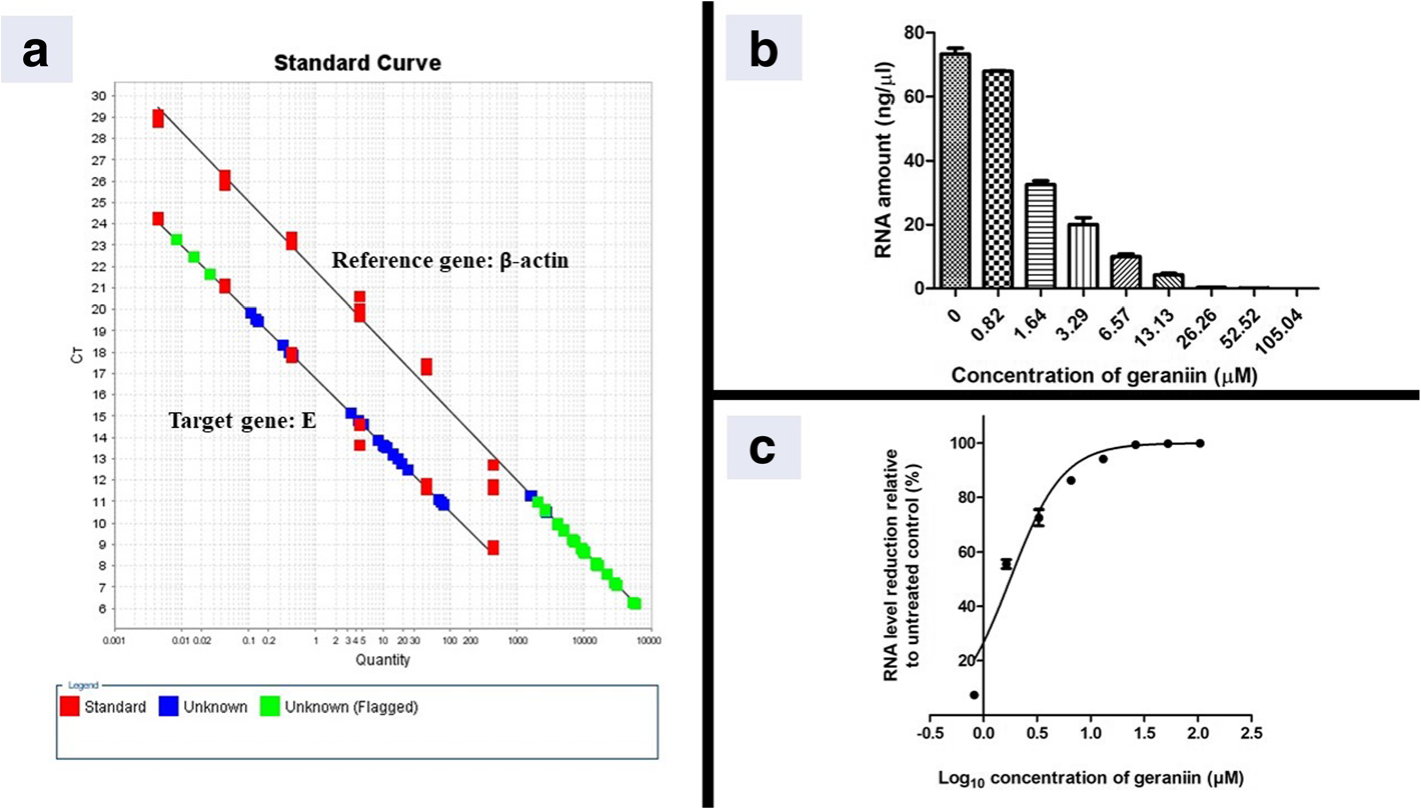
DENV-2 RNA level reduction in infected Vero cells after treatment with geraniin. The DENV-2 RNA production levels in infected Vero cells were quantified by SYBR Green dye-based qRT-PCR. **a** Standard curves generated using serial 10-fold dilutions of stock DENV-2 RNA with known amount of virus. The amplification efficiencies are 109 and 102% for the targeted E gene and the reference gene, β-actin respectively. **b** A complete inhibition of DENV-2 intracellular RNA production was observed after treatment with 26.26–105.04 μM geraniin. **c** A dose-response curve showing concentration-dependent inhibition of DENV-2 RNA production when geraniin was simultaneously added with DENV-2 during adsorption. Data are shown as the mean ± SEM of triplicate from one independent experiment

To investigate geraniin’s effect on DENV-2 RNA synthesis, qRT-PCR was carried out on geraniin-treated and untreated DENV-2 infected Vero cells. Figure 2(a) is a standard curve showing the linear range of SYBR Green fluorescent signal represented by Ct (cycle threshold) value, versus the amount of detected viral RNA for the targeted DENV-2 E gene and the reference gene, β-actin. The amount of detected viral RNA was then plotted against various concentrations of geraniin used in the study. Figure 2(b) shows that when higher concentrations of geraniin were used, the amount of viral RNA detected in infected cells were reduced. The dose-response curve in Fig. 2(c) shows that geraniin inhibits intracellular DENV-2 RNA production after treatment with different concentrations of geraniin. The expression of DENV-2 E gene was completely suppressed when 26.30 μM geraniin was used. The IC_50_ of geraniin is 1.78 μM, as calculated using GraphPad Prism 5 software.

### Effect of geraniin on the spleen of DENV-2 infected BALB/c mice

**Fig. 3:**
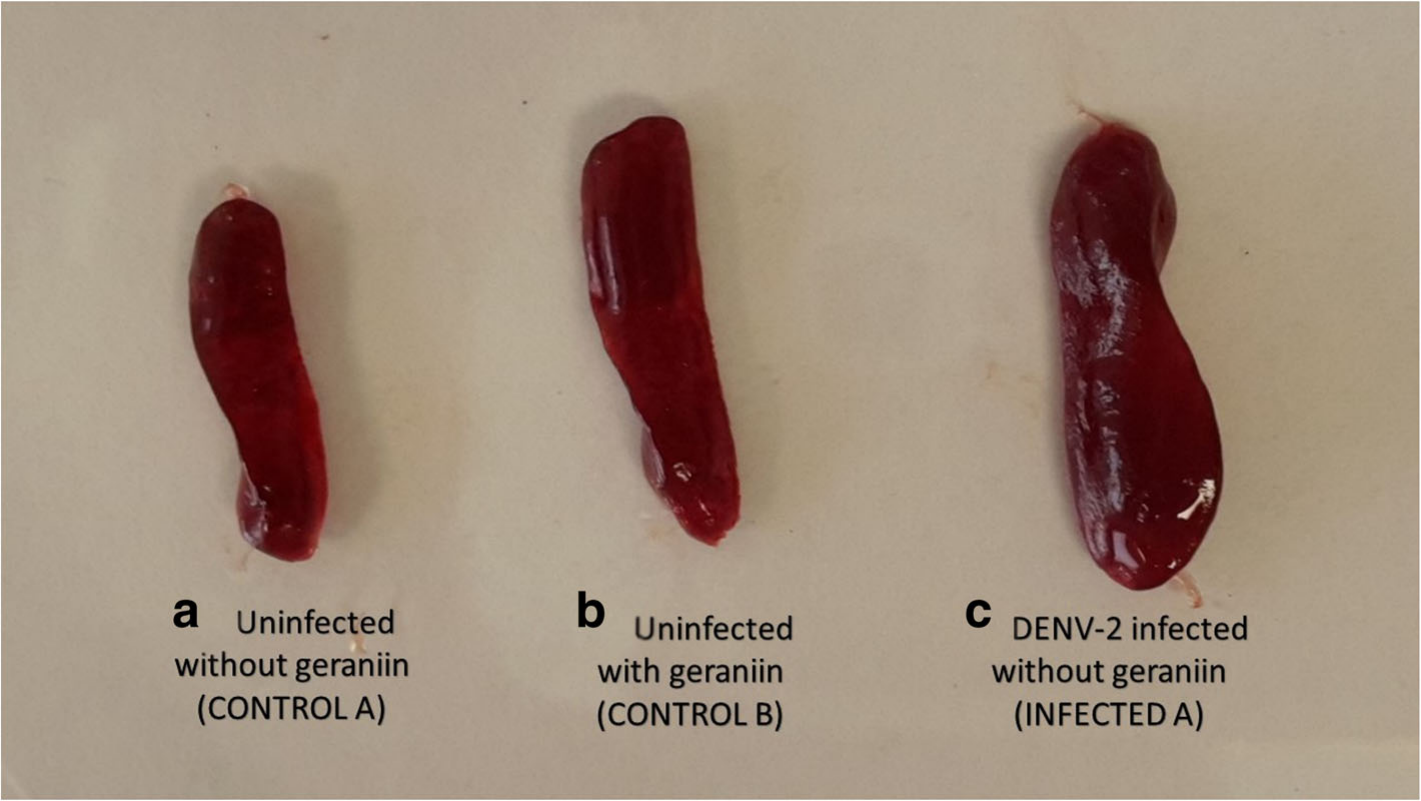
Splenomegaly in DENV-2 infected mice. After eight days p.i., the spleen size of mice from; (**a**) uninfected and untreated (CONTROL A), (**b**) uninfected and geraniin treated (CONTROL B), and (**c**) untreated DENV-2 infected (INFECTED A) groups were photographed for comparison purpose. An obvious spleen enlargement can be seen in all replicates (*n* = 6) of untreated DENV-2 infected mice as compared to the spleen size of those from the CONTROL A and CONTROL B groups

**Fig. 4:**
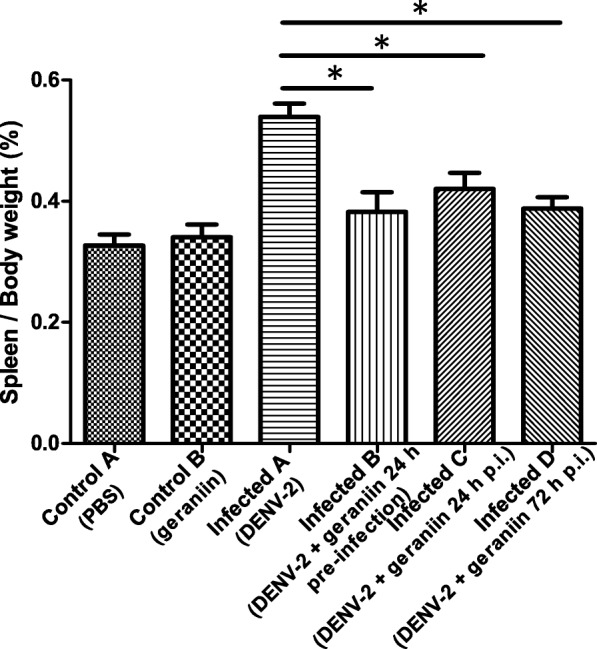
Effect of different geraniin treatments on the spleen of DENV-2 infected mice. The spleen-to-body weight ratio of all three treatment groups (DENV-2 infected and treated with geraniin at 24 h pre-infection, 24 h p.i. and 72 h p.i.), were compared with the untreated DENV-2 infected group after eight days p.i. Data are shown as mean ± SD of six mice per group. Significance of spleen-to-body weight differences was assessed by one-way ANOVA followed by Dunnett’s Multiple Comparison test. Values with statistical significance (*P* < 0.05) are indicated with an asterisk (*)

Throughout the experiment, all mice appeared healthy with no clinical signs of weight loss, ruffled fur or slowing of activity. Spleens of all mice were collected at the end of experimentation and the spleen-to-body weight ratio of each mouse was calculated and the data is presented in Table 2. Spleens of mice from untreated DENV-2 infected group (Infected A) were found to be enlarged and darker in colour compared to those from uninfected and untreated group (Control A) and uninfected and geraniin treated group (Control B). Representative images of the spleen from the three groups were photographed for comparison purposes (Fig. 3). The difference in spleen-to-body weight ratio between control and infected groups are depicted in Fig. 4. There was no significant increase in spleen weight of mice from Control B group when compared to Control A group, indicating that the administration of geraniin did not cause splenomegaly in mice. Splenomegaly was observed in untreated DENV-2 infected mice as the spleen weight in Infected A group was markedly increased compared with that in Control A group. The spleen weight of mice from the three treatment groups (24 h pre-infection [Infected B], 24 h p.i. [Infected C] and 72 h p.i. [Infected D]) were compared against the Infected A group. The increase in spleen weight of mice from the three treatment groups were not as evident as those from the Infected A group. This shows that the administration of geraniin might have reduced or prevented splenomegaly in DENV-2 infected mice.

**Table 2:** Spleen to body weight ratio of each mouse in the different treatment groups

Mouse	Ratio of spleen-to-body weight					
	Uninfected and untreated (Control A)	Uninfected and geraniin treated (Control B)	Untreated DENV-2 infected (Infected A)	DENV-2 with geraniin at 24 h pre-infection (Infected B)	DENV-2 with geraniin at 24 h p.i. (Infected C)	DENV-2 with geraniin at 72 h p.i. (Infected D)
1	0.27	0.33	0.61	0.28	0.32	0.43
2	0.32	0.34	0.55	0.34	0.42	0.38
3	0.33	0.34	0.46	0.47	0.48	0.39
4	0.29	0.26	0.55	0.35	0.44	0.30
5	0.35	0.42	0.49	0.38	0.43	0.42
6	0.40	0.36	0.58	0.49	0.30	0.41
Mean	0.33	0.34	0.54	0.38	0.40	0.39

The body weight of each mouse was measured on the 8th day p.i. while the weight of each spleen was measured after the mice were sacrificed by cardiac puncture. The ratio of spleen-to-body weight of each mouse from the different groups were then calculated. The mean spleen-to-body weight ratio from six replicates mice were used to construct a bar chart of the effect of different geraniin treatments on the spleen of DENV-2 infected mice

### Effect of geraniin on DENV-2 RNA load in infected BALB/c mice

**Fig. 5:**
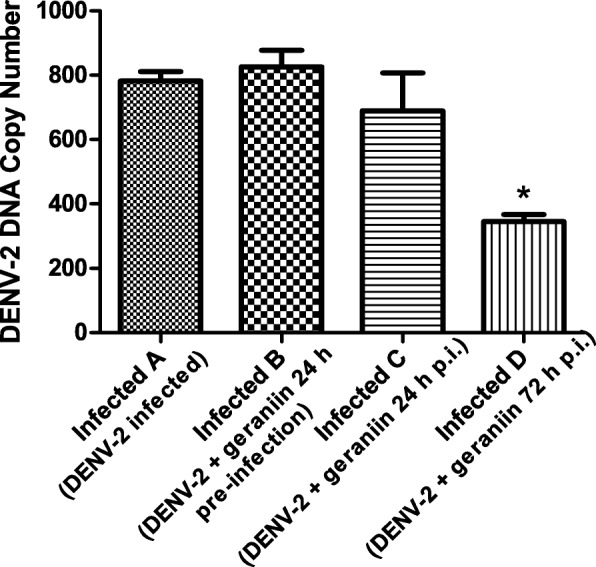
DENV-2 RNA load in mice serum at 8 days p.i. with different geraniin treatments. A reduction in DENV-2 RNA load of infected mice can only be seen when geraniin was administered at 72 h p.i. The difference in RNA load between untreated DENV-2 infected and infected mice administered with geraniin at 72 h p.i is statistically significant (P < 0.05), indicated with the asterisk (*), while it is not significant in the other two treatment groups, as determined by one-way ANOVA followed by Dunnett’s Multiple Comparison test

DENV-2 RNA was extracted from serum collected during cardiac puncture at 8 days p.i., and RT-PCR was carried out to obtain cDNA. The cDNA was then used in qPCR to quantify the RNA load in DENV-2 infected mice. The DNA copy number (RNA load) in serum of each mouse infected with DENV-2 is presented in Table 3 while the difference between the three temporal treatment groups as compared to the untreated DENV-2 infected group is depicted in Fig. 5. It is evident that DENV-2 RNA load in the group treated with geraniin at 72 h p.i. was significantly lower than the untreated DENV-2 infected group (*P* = 0.0002), while no significant difference in RNA load was observed in the other two treatment groups (24 h pre- and 24 h p. i.).

**Table 3:** DENV-2 DNA copy number (RNA load) in serum of each mouse in the different treatment groups

Mouse	DENV-2 DNA copy number			
	Untreated DENV-2 Infected	DENV-2 with geraniin at 24 h pre-infection	DENV-2 with geraniin at 24 h p.i.	DENV-2 with geraniin at 72 h p.i.
1	714.22	938.59	627.82	356.29
2	733.93	891.58	661.59	418.22
3	720.78	769.00	982.43	361.58
4	848.58	654.53	406.89	276.09
5	884.02	736.50	1077.83	373.15
6	791.01	967.98	381.66	284.72
Mean	782.09	826.36	689.71	345.01

The DENV-2 RNA load in serum collected on day 8 p.i. was measured using quantitative RT-PCR. The mean DENV-2 DNA copy number from six replicates mice were used to construct a bar chart of the DENV-2 RNA load in serum of different geraniin treatment groups

### Effect of geraniin on liver pathology of DENV-2 infected BALB/c mice

**Fig. 6:**
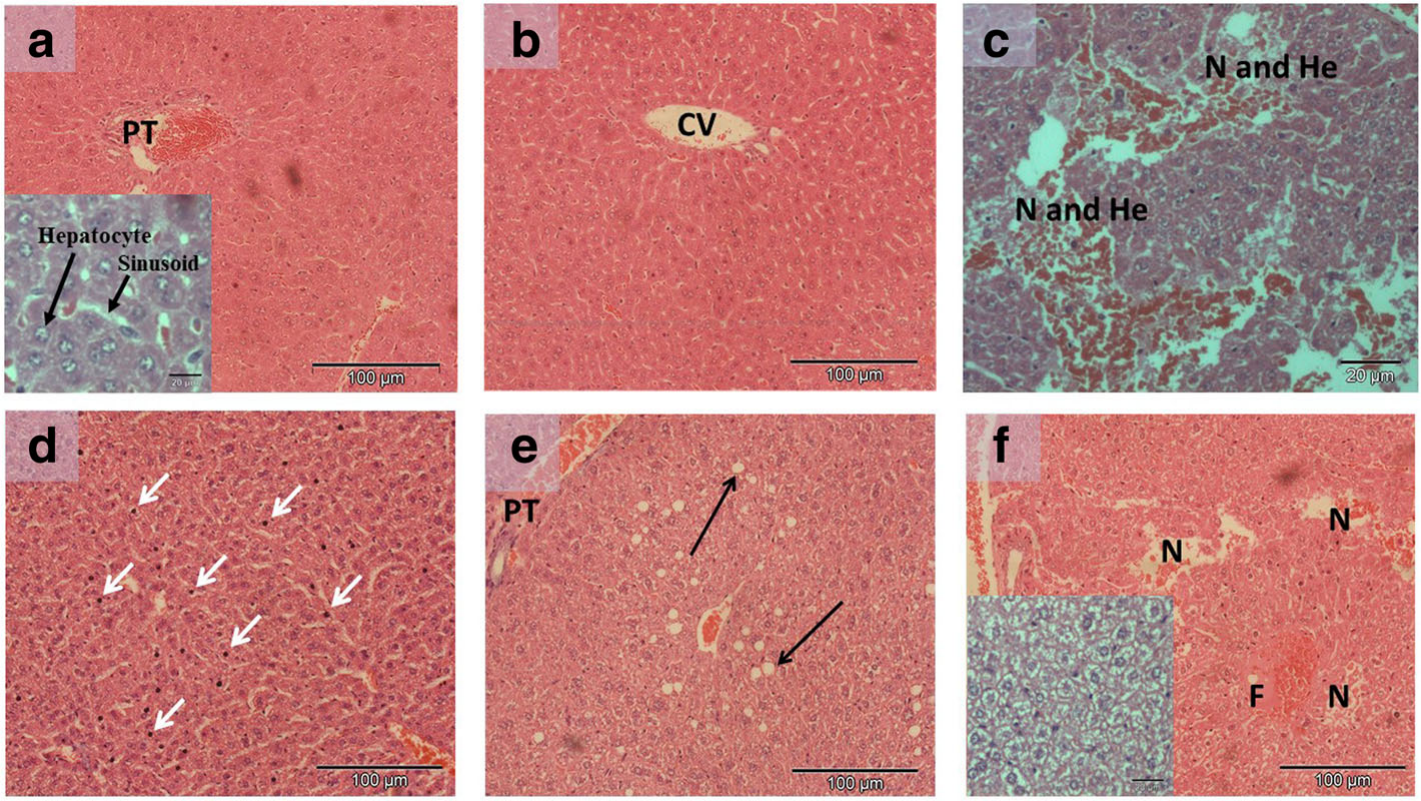
Haematoxylin and eosin staining of mice livers. All mice were euthanized at 8 days p.i. and livers were harvested. **a** Liver showing normal overall architecture of uninfected and untreated mice. Inset: the sinusoids can be seen as pale stained spaces between plates of hepatocytes. **b** Liver of mice where geraniin was given at various time in synchrony with mice that were infected with DENV-2, that is; 24 h pre-infection, 24 h p.i. and 72 h p.i. All DENV-2-uninfected livers that were treated with geraniin showed normal overall liver architecture. **c** Liver of mice infected with DENV-2 act as a virus control to the three treatment groups. Picture shows damaged and degenerated hepatocytes with focal necrosis (N) and haemorrhages (He). **d** Liver of mice treated with geraniin 24 h before infected with DENV-2. Dilatation of sinusoids (enlarged pale spaces in between the hepatocytes) and apoptotic and pyknotic nucleus indicating death of cells (indicated by white arrows) were observed. Generally, hepatocytes seemed normal with slight degenerative changes. **e** Liver of mice infected with DENV-2 and treated with geraniin at 24 h p.i. Moderate hydropic swelling with zonal steatosis i.e. accumulation of triglycerides in hepatocytes (indicated by black arrows). **f** Liver of mice infected with DENV-2 and treated with geraniin at 72 h p.i. Severe generalized or diffuse hydropic swelling of hepatocytes with multifocal and focal necrosis of some parts of the liver was observed, while the blood vessels seemed congested with focal infiltration of fibrin (F) into the parenchyma. Inset picture shows the hydropic degeneration. CV, central vein; PT, portal tract

All mice inoculated with DENV-2 survived the infection without apparent sign of the disease and remained visibly healthy throughout the experiment. Morphological changes in the liver of DENV-2 infected mice were evaluated, and the scoring results are given in Table 4. The histopathological alterations in livers of infected mice treated with geraniin 24 h pre-infection, 24 h p.i. and 72 h p.i. were compared against the alterations in livers of untreated DENV-2 infected group. Hydropic degeneration and venous congestion were observed in all mice infected with DENV-2, regardless of treated or untreated. Interestingly, when geraniin was given at 24 h before or 24 h after infection, there were no areas of necrosis and haemorrhage observed. Contrastingly, the degree of necrosis and haemorrhage observed in livers of mice that were given geraniin at 72 h p.i. was almost comparable to that of untreated DENV-2 infected mice. All assessed histopathological alterations were observed in the untreated DENV-2 infected mice (Infected A) while certain alterations were absent in DENV-2 infected mice treated with geraniin at the three temporal treatment groups (Infected B, C and D). Representative micrographs showing the differences between liver sections of control mice and DENV-2 infected mice with or without geraniin treatments are shown in Fig. 6. Livers of Control A mice, inoculated only with PBS, showed regular structured hepatic parenchyma and the sinusoids capillaries displayed normal endothelial cells [Fig. 6(a)]. In geraniin treated mice (Control B), that were administered with a similar dose of geraniin as the treatment groups, the liver histology [Fig. 6(b)] was comparable to that of the Control A group. Severe hepatic injury manifesting as multifocal hepatic necrosis and haemorrhages, pyknotic necrosis of cells, diffuse microsteatosis, diffuse hydropic degeneration and dilatation of sinusoids were observed in the liver sections of all untreated DENV-2 infected mice [Fig. 6(c)]. However, when geraniin was administered at 24 h pre-infection [Fig. 6(d)] and 24 h p.i. [Fig. 6(e)], we noticed that the liver damages were not as severe as the untreated DENV-2 infected mice, with an absence of necrotic areas and only mild to moderate histopathological lesions. In the 72 h p.i treatment, liver damage due to DENV-2 infection was visible, with moderate to severe multifocal necrosis, haemorrhage and hydropic degeneration of hepatocytes [Fig. 6(f)].

**Table 4:** Semi-quantitative histopathological assessment of the liver sections for DENV-2 infected mice treated with different geraniin treatments

Descriptive parameter	Infected A (DENV-2)	Infected B (DENV-2 + geraniin 24 h pre-infection)	Infected C (DENV-2 + geraniin 24 h p.i.)	Infected D (DENV-2 + geraniin 72 h p.i.)
	Mean ± SD	Mean ± SD	Mean ± SD	Mean ± SD
Hydropic degeneration	2.5 ± 0.5	1.8 ± 1.2	2.7 ± 0.5	3.0 ± 0.0
Venous congestion	3.0 ± 0.0	2.0 ± 0.8	1.0 ± 0.0	2.0 ± 0.0
Necrosis and haemorrhage	2.7 ± 0.5	0.0 ± 0.0	0.0 ± 0.0	2.5 ± 0.5
Steatosis	0.8 ± 0.8	0.0 ± 0.0	1.7 ± 0.5	0.0 ± 0.0
Formation of pyknotic nucleus	3.0 ± 0.0	1.5 ± 0.5	0.0 ± 0.0	0.0 ± 0.0
Average Score	2.4 ± 0.3	1.1 ± 0.5	1.1 ± 0.3	1.5 ± 0.3

Overall, untreated DENV-2 infected mice showed a moderate to severe liver damage with the presence of all assessed histopathological alterations. Livers of infected mice treated with geraniin at 24 h pre-infection and 24 h p.i. showed a mild damage while those treated with geraniin at 72 h p.i. showed a mild to moderate liver damage. Data are expressed as mean and standard deviation (SD)

## Discussion

Compounds with antiviral properties against dengue serve as a promising alternative to dengue therapy. In our previous in vitro study, we showed that geraniin inhibits early stage of DENV-2 replication, particularly the attachment process, by binding to the viral E protein, specifically at the Domain III region [28]. In our current study, we further prove the inhibitory potential of geraniin by quantifying the DNA production in geraniin treated-infected Vero cells. The complete suppression of DENV-2 E gene expression when geraniin was used at higher concentrations to treat infected cells proved that geraniin is effective against DENV-2 in vitro. Based on all our preliminary in-vitro studies, we therefore hypothesised that geraniin binds directly to DENV via the Domain III region of viral E protein thus inhibiting viral entry and replication. In this study, we intend to show the effect of geraniin on inhibiting virus by the reduction in viral RNA load and liver damage in BALB/c mice. The effects of DENV-2 infection on liver and viral RNA load in serum were examined at the eighth day p.i. as the end-point for the study. The eighth day end-point was based on a study by Paes et al., on the effect of DENV-2 on BALB/c mice, where he showed that virus level in serum peaks at 7 days p.i. and subsequently declined at the 14th day p.i., with all mice surviving the infection and not demonstrating any clinical signs [8]. In our study, liver damage and the detection of RNA viral load until 8 days p.i indicated that the DENV-2 is actually replicating in our model.

Although the liver was the main organ examined in this study, gross pathology of the spleen was also analysed as it is a visible marker for virus infection induced inflammatory response [29]. Splenomegaly has been observed in 12–34% of human DENV infections and in other mouse models of DENV [30]. In our study, the splenomegaly observed in DENV-2 infected mice indicates that BALB/c mice have a strong inflammatory response to dengue infection. The spleens sizes and weights of mice treated with geraniin were normal. This may indicate that geraniin itself does not induce inflammatory responses; as also observed in other studies where geraniin was shown to be an anti-inflammatory agent [31–33]. A slight increment was observed in the spleen size of infected mice from the three geraniin treated groups as compared to the uninfected control. However, the increment was not as prominent as in the untreated DENV-2 infected mice. This shows that the administration of geraniin either before or after infection results in a lesser extent of DENV-2-induced splenomegaly.

Unlike the cell culture system where viruses can directly enter cells to replicate and viral inhibitors can almost instantaneously react with their target of action; in vivo systems are more complicated as multiple enzymes and proteins will be present. Administered drugs will be subjected to various pharmacokinetic processes, which determines how rapidly and for how long the drug will appear at the target organ [34]. Most of the times, many drug effects observed in vitro could not be reproduced in vivo as their bioavailability had been reduced, depending on its route of administration. A pharmacokinetic study for the determination of geraniin in rat plasma suggested that geraniin was distributed in vivo rapidly, with an alpha half-life (t_1/2α_) of 0.21 ± 0.10 h for intravenous administration; while its beta half-life (t_1/2β_) was 7.20 ± 2.20 h, suggesting that the elimination of geraniin was slow [35]. This study also revealed that when the rats received oral administration of geraniin, the content of geraniin was not detected in the rats’ plasma. There has been no study that reported on the detection of intact geraniin in the circulation or urine upon oral dosing. Orally administered geraniin would be subjected to extensive degradation and metabolism mainly by the intestinal bacteria, producing various metabolites which are eventually absorbed and stays in the body for a relatively long duration [14]. It is believed that these metabolites may be the key players that account for the bioactivity of geraniin as it was already shown that metabolites play important roles in biological antioxidants after oral administration of intact ellagitannins [36].

Compounds that are injected intravenously have a 100% bioavailability and is the fastest and most certain route of drug administration [34, 37]. Since we administered geraniin intravenously, the absence of DENV-2 in the systemic circulation upon its administration would influence in geraniin’s distribution to other organs or cells where it can bind to its target. Some drugs have an affinity for protein molecules and bind to them while streaming through the bloodstream, which delays the distribution of the drug and its onset of action. Tannins are known for their capability of binding to proteins [38]. The protein binding property of tannins is the major factor that contributed to their various physiological activities. In our circumstances, the ability of the tannin geraniin to bind with DENV-2 viral protein contributes to its anti-dengue activity. When the viral particles are present in the circulation during geraniin’s administration, geraniin can directly binds to the viral protein and prevents further infection. However, if the viral particles are absent, or their numbers are too low for the geraniin to act upon, there is a possibility that geraniin could be bound to other proteins present in the circulation, such as the plasma proteins, thus reducing its efficacy.

The presence of DENV-2 particle during administration of geraniin would enable an interaction to occur and geraniin should be able to exert its effect and prevent virus from replicating. At 72 h p.i., DENV-2 would have been released from its secondary target organs such as the liver and should be circulating in the systemic circulation to infect more cells. Hence, upon geraniin’s administration at 72 h p.i., DENV-2 present in the systemic circulation would bind to geraniin thus preventing DENV-2 from infecting more cells and causing further infection in the mice, as evidenced in the reduction of viral RNA load. The inability of geraniin to effectively exerts its full antiviral potential when it was administered at 24 h pre- and 24 h p.i. could be due to the nature of DENV infection. As infection of fixed macrophages (Kupffer cells) in the liver during pathogenesis of *Flavivirus* occurs 24 h after inoculation [39], it is indisputable that at 24 h p.i., DENV-2 would still be replicating in its secondary target organs and the amount of viral particle present in the systemic circulation would be minimal. Thus, geraniin administered at 24 h p.i. could not bind to its target. Since geraniin is distributed rapidly in vivo, it would no longer be in the circulation by the time DENV-2 was released into the bloodstream. Likewise, when it was administered 24 h pre-infection, geraniin would not be in circulation by the time of DENV-2 inoculation into the mice, resulting in secondary viral replication in various organs, thus the high RNA load observed by the end of the 8th day p.i.

Nevertheless, this finding does not imply that geraniin treatment at 24 h pre-infection and 24 h p.i. is totally ineffective in reducing DENV-2 RNA load as this may be overcome by administrating multiple or daily dosages of geraniin; something worth exploring in future studies. The additional geraniin doses could replenish the amount of geraniin present in the systemic circulation, resulting in the ability to exert its effect on DENV-2 when released into the bloodstream. Various regimens of prophylactic drugs dosing can be used to prevent certain diseases especially by travellers who are going to countries where that certain disease is endemic. For instance, several options for malaria chemoprophylaxis exist such as chloroquine and mefloquine [40]. A more extensive animal study that includes testing different dosages of geraniin, multiple or daily dosing before (prophylactic) and after infection may need to be conducted in the future.

Based on previous studies [8, 41, 42] that used BALB/c mice as a model for dengue infection, liver was among the first organs to be infected and hepatic injury was seen as early as 2 days p.i. Thus, liver injury is typical in all mice infected with DENV-2. Severe liver injury with histopathological changes such as hepatocyte swelling and vacuolization, necrosis, and steatosis can be seen in liver infected by DENV [43]. In a study conducted by Paes and his team, hepatic injury in DENV-2 infected BALB/c mice was observed as early as 2 days p.i., and at the 3rd day p.i., hepatocytes showed diffused steatosis in midzonal areas, while at 7 days p.i., necrosis and a strong flux of oedema was observed [8]. Regeneration of hepatocytes was only observed after the 17th day p.i. In our study, the histopathological changes observed in the liver of DENV-2 infected mice were consistent with the findings in other studies [8, 42, 43]. Despite a reduction in DENV-2 RNA load when geraniin was given at 72 h p.i., the histopathological changes observed in this treatment group were almost similar to the lesions observed in untreated DENV-2 infected mice; suggesting that the extensive damage seen to the liver by this time was not reversible by geraniin or its metabolites. Whilst treatments of geraniin at 24 h pre- and 24 h p.i. displayed substantial liver protection, delayed administration of geraniin at 72 h p.i. may not protect against severe liver damage even though it reduces the RNA load in DENV-2 infected BALB/c mice. These findings therefore imply that the administration of geraniin before infection or before the occurrence of severe liver injury may help in reducing liver damage and may assist in liver regeneration process. The reduced severity in liver injury may be attributed to the ability of geraniin and its metabolites to protect against liver damage, as it has been previously proven that these compounds can reduce various types of liver injury [44–47].

In DENV infection, there is a relationship between disease severity and the virus load [24]. Results from clinical studies showed that the quantity of virus circulating in blood of patients who develop severe dengue is higher by around 1–2 logs compared with patients suffering from mild dengue disease [23]. The reduction in DENV-2 RNA load when geraniin was administered at 72 h p.i. suggests the potential benefit of geraniin as an antiviral therapy that can reduce viral load in the acute phase of the disease. Previous studies have shown the presence of DENV during the post-defervescence period in patients with severe dengue [48]. These findings indicate that the clinical manifestations of severe dengue may, in part, be virus-driven and support the hypothesis that antivirals given at later stages of illness may still be beneficial. Current treatment for severe cases is mainly supportive involving intravenous fluid replacement, which is crucial in preventing impending shock that may lead to death. Since we had shown that geraniin can reduce DENV-2 RNA load, geraniin might have the potential to be developed as a therapeutic agent for DENV infections and possibly reduce progression to severe dengue when administered together with intravenous fluid. Although oral administration is considered to be the most acceptable and economical method of administration [49], the low bioavailability of geraniin might hinder its development as an oral drug. This low bioavailability of orally administered geraniin is mainly due to its large molecular size and low solubility in gastric fluid, which becomes a big challenge for its clinical application [14]. Therefore, the bioactivities that follows oral intake of geraniin are mostly attributed to its smaller metabolites, suggesting that these compounds share a common intrinsic trait which is unaffected by the degradation of geraniin. Hence, investigation on the anti-dengue activity of geraniin’s metabolites is important towards revealing the exact mechanism behind geraniin’s inhibitory potential and to its development as a treatment for dengue infection.

## Conclusions

All in all, our study is yet a preliminary one, aimed at investigating the efficacy of geraniin in a small animal model and to prove that the inhibition of DENV-2 in cell culture could still be observed in vivo. Further work using interferon deficient mice models that can replicate the true nature of dengue pathogenesis is imperative so that the potential of geraniin as DENV inhibitor could be truly verified. Nevertheless, this study paved the way towards future work involving further understanding of the detailed knowledge on pharmacokinetic disposition of geraniin, to ensure safety and tolerance as well as to predict its optimum dosage and usefulness before it can be effectively used in dengue therapy.

## Abbreviations

ATCC: American type culture collection
CO_2_: Carbon dioxide
DENV: Dengue virus
DENV-1: Dengue virus type-1
DENV-2: Dengue virus type-2
DENV-3: Dengue virus type-3
DENV-4: Dengue virus type-4
DMSO: Dimethyl sulfoxide
E: Envelope
FBS: Fetal bovine serum
h: Hour
HEPES: 4-(2-hydroxyethyl)-1-piperazineethanesulfonic acid
IC_50_: Half maximal inhibitory concentration
MEM: Minimum essential medium
min: Minute
p.i.: Post-infection
PBS: Phosphate buffered saline
qRT-PCR: Quantitative real time RT-PCR
RT-PCR: Reverse transcription polymerase chain reaction
